# Effects of Mn addittion, cooling rate and holding temperature on the modification and purification of iron-rich compounds in AlSi10MnMg(Fe) alloy

**DOI:** 10.1016/j.heliyon.2023.e13005

**Published:** 2023-01-21

**Authors:** Jon Mikel Sanchez, Maribel Arribas, Haize Galarraga, Maider Garcia de Cortazar, Marco Ellero, Franck Girot

**Affiliations:** a TECNALIA, Basque Research and Technology Alliance (BRTA), Astondo Bidea E-700, 48160, Derio, Spain; b Basque Center for Applied Mathematics, Alameda de Mazarredo 14, 48400, Bilbao, Spain; c Ikerbasque, Basque Foundation for Science, María Díaz de Haro 3, 48013, Bilbao, Spain; d Zienkiewicz Centre for Computational Engineering, Swansea University, Swansea, SA1 8QQ, UK; e Department of Mechanical Engineering, Engineering School of Bizkaia, University of the Basque Country (UPV/EHU), Alameda de Urquijo s/n, 48013, Bilbao, Spain

**Keywords:** Aluminum alloys, Circular economy, Fe-rich phases, CALPHAD, Recycling, Precipitation, Solidification, Sedimentation

## Abstract

The use of secondary aluminum alloys in industry is still limited by the high Fe contents in recycled alloys. In general, the Fe-rich intermetallic compounds deteriorate the performance of secondary Al–Si alloys, specially the β-Fe phase. To mitigate the detrimental effects of iron, the influence of diferent cooling rates and holding temperatures on the modification and purification of iron-rich compounds in commercial AlSi10MnMg alloy with 1.1 wt % Fe was studied. According to the results obtained by CALPHAD calculations, the alloy was modified by adding a 0.7 wt%, 1.2 wt%. and 2.0 wt% of Mn. The phase formation and morphology of iron-rich compounds was systematically studied and correlated by different microstructural characterization techniques. The experimental results showed that the detrimental β-Fe phase can be avoided by adding at least 1.2 wt % of Mn at the studied cooling rates. Finally, the effect of different holding temperatures in the sedimentation of Fe-rich compounds also was studied. Hence, the gravitational sedimentation experiments at different holding times and temperatures were conducted to validate the feasibility of the methodology in different processing conditions. The experimental results showed a high Fe removal efficiency up to 64% and 61%, after a holding time of 30 min at 600 °C and 670 °C, respectively. The addition of Mn improved the Fe removal efficiency but not gradually, as the best results were obtained in the alloy containing 1.2 wt % Mn.

## 1 Introduction

The circular economy is the new paradigm to pivot towards sustainable social and economic development, a challenge, or rather an urgent need of the society [1,2]. Nowadays, a great deal of effort is being made in the development of sustainable materials with improved properties, avoiding scarce, strategic, or hazardous elements, and high energy consumption processes. Secondary or recycled aluminum is considered one of the most promising engineering alloys towards a sustainable economy, due to increasing demand for different applications and because the increasing of available aluminum scrap in the future [3]. With a relative low density compared to other metals, aluminum alloys are used as lightweight structural material, offering the possibility of reducing greenhouse gas emissions in the transportation industry. Furthermore, in the irreversible and compulsory process of decarbonization of the society, aluminum alloys are emerging as key materials for storage and transportation of liquid hydrogen [4,5,6].

Primary aluminum alloy manufacturing from ores or minerals is a very energy intensive process. However, secondary aluminum production from recycled scrap only needs 5% of such energy, becoming a material with considerably higher sustainability [7,8,9]. A large amount of aluminum alloy scrap is post-consumer scrap, with high content of impurities and contamination, representing a considerable challenge for the recyclability of this material. To increase the recyclability of aluminum alloys, impurities must be reduced to an acceptable level. Among others, Fe is a common impurity due to its gradual accumulation during the production of aluminum alloys [10]. This is caused by the fact that the solubility of Fe is below 0.05 wt% in solid aluminum [11]. In general, Fe is related to a negative effect in the mechanical properties in recycled aluminum alloys. Specifically, the ductility decreases with the increasing of Fe content [12–14]. Taylor et al. described the formation of Fe-rich compounds during solidification, and how these phases can adversely affect the ductility [15]. On the other hand, some benefits were also reported. For example, Zhu et al. reported that the addition of Fe can enhance yield and ultimate tensile strength in Al–Mg and Al–Mg–Mn alloys [16]. Lin et al. observed that the addition of Fe improves mechanical properties at elevated temperatures [17]. According to Zhao et al., the addition of Fe can reduce the die soldering in die casting processes of Al–Mg–Mn–Fe alloy [18].

Al–Si alloys constitute 80% of the aluminum casting alloys employed in the manufacturing industry [19]. Therefore, Fe-rich phase formation, detrimental effects on the mechanical properties, and methods to modify and/or remove Fe-rich phases have been extensively studied for Al–Si casting alloys. Regarding phase formation, Zhang et al. reviewed the formation of Fe-rich intermetallic compounds in Al–Si alloys. According to this work, the most common phases in Al–Fe–Si systems are ternary α-AlFeSi (α-Fe) and β-AlFeSi (β-Fe) phases [13]. Among these, the β-Fe phase is considered the most harmful phase to the mechanical properties of the alloy due to its brittleness and morphology [18,[20], [21], [22], [23]]. This phase presents a needle-like morphology when observed in a two-dimensional micrography, even though the β-Fe phase has a platelet-like morphology in three dimensions [15]. Hence, in this work a platelet-like morphology is considered for this phase. By contrast, the α-Fe phase shows a more compact morphology, and this phase is considered less detrimental to the mechanical properties. Depending on the solidification sequence α-Fe phase shows different morphology. If it solidifies as predendritic or primary compound, it presents a star-like (2D view) or polygonal morphology (3D view) and is commonly known as “sludge” [24,25]. Instead, if it solidifies as post-dendritic compound, α-Fe phase exhibits Chinese-script morphology, and grow along the interdendritic region. Therefore, the morphology of the α-Fe compounds changes from the faceted polyhedron (polygonal) to a convoluted eutectic structure (Chinese-script) once α-Al dendrites begin to form [26].

Fe-rich intermetallic phases affect the performance of the alloys, which is generally controlled by their crystal structure, size, morphology and volume fraction of the compounds [27]. The crystallographic properties and the nominal composition of the most common Fe-rich intermetallic phases were summarized in Ref. [25]. Metallurgy of Fe-rich intermetallic phases become more complex as the number of elements in the alloy are increased. Depending on the secondary element contents, such as Mn, Cr, etc., crystallography, and morphology of Fe-rich intermetallic phases can be modified [18,23,[28], [29], [30], [31], [32], [33], [34]]. Some authors studied the effects of the addition of different elements to modify the platelet-like morphology β-Fe phase to the less detrimental α-Fe phase [18,31,35,36]. In particular, the addition of Mn modified the morphology of β-Fe-rich phase from the brittle platelet to more compact morphologies (α-Fe) restoring the tensile strength and elongation [23,33,37,38]. Nevertheless, the total amount of Mn required to avoid the formation of β-phase is difficult to determine, since it is affected by many factors, such as cooling rate (CR), Si and Fe content [13,30].

Morphology and size of Fe-rich phases is also controlled by the CR-s of different manufacturing processes. Fe-rich phases solidify as primary, pre-eutectic, co-eutectic, and post-eutectic intermetallic phases at different stages of solidification through predendritic, eutectic, and peritectic reactions [39]. Hence, controlling the CR and solidification properties is also very important in the formation of Fe-rich detrimental phases of secondary aluminum alloys [21]. Tang et al. observed that a high CR result in finer β phase in Al–7Si alloy processed by permanent mold casting [40]. Liu et al. determined that the high CR of high pressure die casting process, avoided the formation of β-Fe phase in AlSi9Cu3 (A383) alloy with a Fe content of 1 wt% [25]. Recently, Lan et al. developed Al–Fe–Si alloys by adjusting elemental composition and CR, the tensile properties of the developed Al–5Fe–3Si alloy were like those of commercial Al–8Si-0.3Mg-0.2Mn-0.2Fe (A356) alloy [41]. In the same work, it was also pointed out that decreasing the Si content, β-Fe phase transformed to α-Fe phase and Al_13_Fe_4_, and the overall volume fraction of the Fe-rich phases decreased. Seifeddine et al. observed that the different CR of the manufacturing processes also influence on the volume fraction, size, and morphology of α-phase. In this regard, Liu et al. [25] reported that the greatest influence in AlSi9Cu3 alloy was obtained in the samples solidified at 1–10 K/s range, observing a reduction of the fraction and size of primary α-phase with an increase of the CR.

The above-mentioned elemental modification and control of the solidification properties are useful approaches to reduce the detrimental effects of Fe-rich phases. The above-mentioned elemental modification and control of the CR of the different manufacturing processes, are useful approaches to reduce the detrimental effects of Fe-rich phases. However, the total amount of Fe in the alloy cannot be reduced. In fact, the total volume of Fe-rich phases increases with the addition of Mn, Cr, etc., and gradually deteriorates the properties of the alloys, even after the elemental modification treatment. To overcome this inconvenience, there are different techniques to decrease the Fe concentration from molten aluminum. Most of those methods are based in the formation of primary Fe-rich phases by proper Mn or combined Mn and Cr additions [32]. Then, the removal of these phases can be performed by different techniques. Zhang et al. reviewed several technologies to remove Fe from aluminum alloys, concluding that dense phases can be removed by gravitational sedimentation, electromagnetic separation, or centrifugation methods [13]. Elimination of Fe by gravitational sedimentation was proved as an efficient method for high-Si content aluminum alloys [42]. In this approach, the melt is hold between the formation temperature of predendritic Fe-rich compounds and α-Al phase. The Fe-rich compounds segregate to the bottom of the melt due to the higher density, reducing the Fe content. Donk et al. observed that α-Fe phase segregated immediately during the cooling from 840 °C to a holding temperature of 600 °C [43]. Scamans et al. also observed that Fe-rich intermetallics started to precipitate immediately when melting temperature decreased below the liquidus [44]. However, the gravitational sedimentation could require long holding times, and specific holding temperatures below α-Fe and the formation temperature of α-Al phase, which are not always employed in the manufacturing process. For example, in the case of the casting process of AlSi10MnMg alloy, the recommended pouring temperature is about 680 °C–710 °C [45]. This is significantly above the liquidus temperature, so no sedimentation occurs, and this pouring temperature difference makes the sedimentation process ineffective [30].

Generally, elemental modification of Fe containing Al–Si alloys was made based on recommended Fe:Mn or (Fe,Mn):Si ratios [25,34,39,46]. For example, it was observed that the formation of detrimental β-Fe phase was avoided in Al–Mg–Si–Mn and Al–Mg–Si diecast alloys when Mn:Fe ratio is greater than 0.5 [12]. Other authors considered the so-called sludge factor, which predicts the tendency to form primary α-Fe phase considering the Fe, Mn and Cr contents [34,47]. But the recommended parameters were not always sufficient conditions to avoid the formation of β-Fe compounds, or to define the optimum process conditions to form primary α-Fe phase [47].

Recently, Chen et al. demonstrated that CALPHAD-based tools are very useful for the application of recycling, since they can be used in the elemental modification and process optimization of secondary aluminium alloys [48]. This tool can handle with the complexity of phase formation in the multicomponent systems of recycled aluminium alloys at different temperatures. Consequently, it can be applied in the recycling of materials by estimating the total amount of corrector elements required during casting. CALPHAD-based software was successfully employed to avoid the formation of undesirable Fe-rich compounds by elemental modification in Al–7Si–3Cu cast alloy [29], and in Al–Mg–Mn–Fe alloys processed by squeeze casting without heat treatment [18]. The mechanical properties of Al–Mg–Si–Fe alloys also were improved by the proper addition of Mn calculated by CALPHAD [12]. Recently, Sree et al. improved the direct chill casting process of A6082 alloy by CALPHAD calculations [49]. In their work, the most efficient processing temperatures were predicted by CALPHAD techniques.

In this study, 1.1 wt% of Fe was added in a hypoeutectic AlSi10MnMg (A365) alloy, developing experimental alloys with a composition of recycled cast aluminum alloys. According to the results obtained by CALPHAD methodology, the alloy was modified by a variable content of Mn. The effects of different CR in the phase formation of Fe-rich phases were studied. Finally, gravitational sedimentation at different holding temperatures and times were conducted to purify the alloy.

## 2 Experimental methods

### 2.1 Thermodynamic modelling of the alloys

**Fig. 1:**
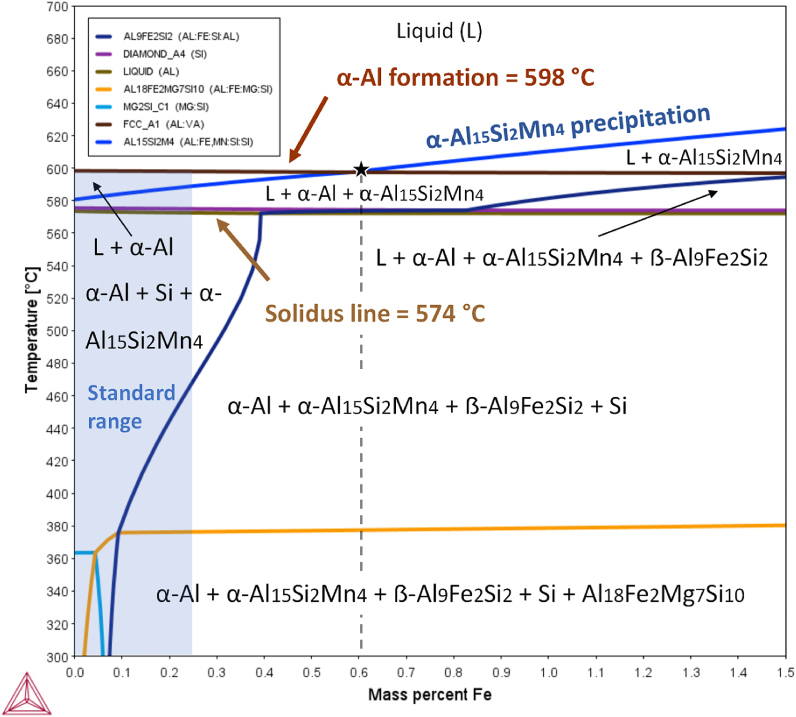
Calculated AlSi10MnMg isopleth and varying Fe content in wt. %.

The software Thermo-Calc version 2021b (Thermo-Calc Software AB, Stockholm, Sweden) in conjunction with the TCAL8 thermodynamic database was used for calculations of the equilibrium and non-equilibrium phases as a function of temperature [50]. These calculations supported the definition of the elemental modification performed to avoid the formation of β-Fe phase and define the most efficient temperatures for the sedimentation of primary α-phase in AlSi10MnXMg alloy containing 1.1 wt% Fe. Fig. 1 shows the equilibrium phase diagram as a function of temperature of AlSi10MnMg alloy with a variable content of Fe in the range 0–1.5 wt%. The representative chemical composition considered in this calculation was 9.5 wt% Si, 0.5 wt% Mn, 0.1 wt% Mg and balanced Al. The stable Fe-rich phases at high temperatures are designated as Al15Si2M4 and Al9Fe2Si2 by Thermo-Calc, corresponding to cubic α-Al_15_Si_2_(Fe,Mn)_4_ or α-Fe and monoclinic β-Al_9_Fe_2_Si_2_ or β-Fe phases, respectively.

According to ENAC–43500 the Fe composition of the alloy must be under 0.25 wt%, this composition was highlighted in the isopleth in blue. As can be seen in the isopleth, this chemical composition avoids the formation of β-Fe phase. The equilibrium phase diagram also shows that the formation of α-Al phase occurred at 598 °C. Therefore, the first phase to solidify is α-Al phase, followed by the precipitation of post-dendritic α-Fe phase and Si, respectively. There is also an important point in the diagram at 0.6 wt% Fe and 598 °C. At this point, the eutectic reaction of α-Fe phase occurs, which means that under a concentration below 0.6 wt% Fe, all the Fe-rich phases solidifies as postdendritic intermetallic compounds. Therefore, in AlSi10MnMg(Fe) alloy the melt purification of Fe by gravitational sedimentation is not feasible in compositions below 0.6 wt% Fe. On the other hand, with a concentration above 0.6 wt% Fe, α-Fe-rich phases solidifies through predendritic solidification reaction, forming primary α-Fe phase. The temperature interval between liquidus and α-Al in the range of 0.6 wt% to 1.5 wt% Fe is very narrow, especially at the lowest content of Fe. Furthermore, the maximum α-Fe phase formation temperature in the diagram is 621 °C. According to the results of CALPHAD calculations, Fe-rich phases can be removed when the melt is maintained at a temperature below 621 °C and above the temperature of α-Al formation (598 °C). A holding temperature of 600 °C was successfully employed to remove Fe in the direct chill casting of A380 alloy with accumulated concentration of Fe and Mn [44]. Instead, in some casting processes, such as high pressure or gravity die casting, this temperature can be significantly lower than the required pouring temperature to fill the mold, making the gravitational sedimentation inviable. The latter means that elemental modification of the alloy is required to modify primary α-phase formation temperature and enable sedimentation. The influence of Fe content on the formation of detrimental β-phase is also observed in the phase diagram. According to thermodynamic calculations, the formation temperature of β-phase is higher than the solidus temperature when the Fe content is above 0.85 wt%, and continuously increases almost reaching the α-Al formation temperature when the Fe content is 1.5 wt%. This means that all the stabilized β-phase precipitates as post-dendritic compound for the entire composition considered.

**Fig. 2:**
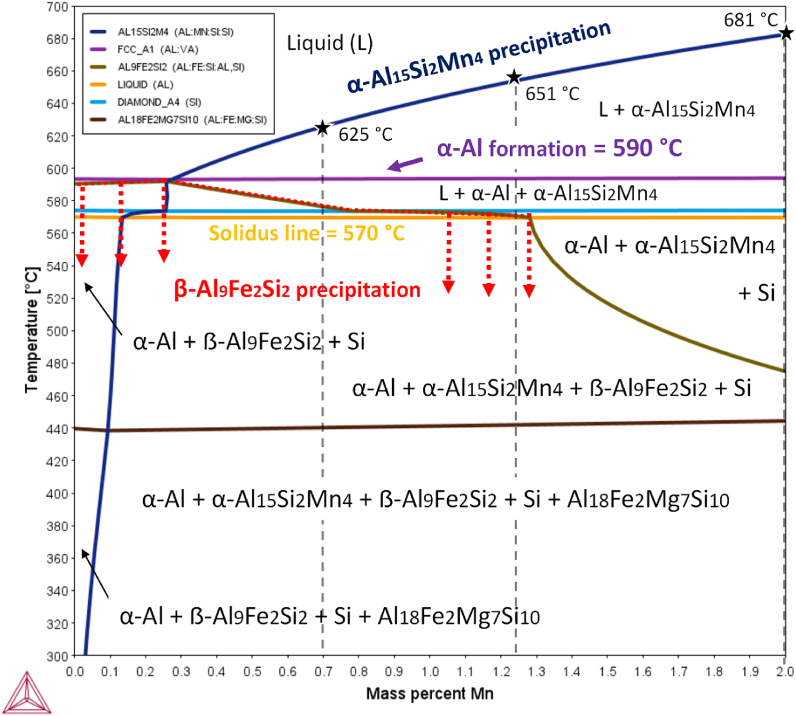
Calculated AlSi10MnMg isopleth with 1.1 wt% Fe and varying Mn content in wt.%. The selected alloy compositions are denoted.

In Fig. 2 the effect of the addition of Mn (up to 2 wt%) in AlSi10MnMg(Fe) alloy containing 1.1 wt% Fe in the equilibrium phase diagram is plotted. The equilibrium phase diagram shows that the formation of α-Al phase occurred at 590 °C over the entire range studied, which is 2 °C above the formation of α-Al phase in AlSi10MnMg(Fe) alloy in Fig. 1. The isopleth also shows that the addition of Mn promotes the formation of pre-dendritic α-Al_15_(Fe,Mn)_4_Si_2_ phase, when the amount of Mn is over 0.3 wt%, which means that precipitation of Fe-rich compounds cannot occur under this range. When the amount of Mn is above this quantity, the solidification temperature of α-Al_15_(Fe,Mn)_4_Si_2_ increases progressively, showing a temperature interval for sedimentation wider than that shown in Fig. 1. The maximum precipitation temperature is 681 °C at 2.0 wt% of Mn, which means that the sedimentation can occur at the recommended pouring temperatures of AlSi10MnMg alloy (680–710 °C). Below solidus line, there are three regions depending on Mn content that should be distinguished. The first one, is the region with a composition of Mn below 0.15 wt%, where the equilibrium phases are α-Al, post-dendritic β-Al_9_Fe_2_Si_2_ and Si phases. This region is not of interest, as there is not the possibility of Fe removal by gravitational sedimentation is not feasible. Furthermore, the low quantity of Mn is not enough to promote the formation of primary α-Fe phase. Therefore, the precipitation of β-phase is not avoided. The second one, is the region with a composition between 0.15 and 1.29 wt% Mn. The equilibrium phases are α-Al, α-Al_15_(Fe,Mn)_4_Si_2_, β-Al_9_Fe_2_Si_2_ and Si phases. At this composition, sedimentation is expected to occur, what it is interesting as α-Fe phase precipitates at relatively high temperatures. However, in this range the formation of β-phase still occurs. Finally, when the amount of Mn is above 1.29 wt%, the stable phases are α-Al, α-Al_15_(Fe,Mn)_4_Si_2_, and Si. The addition of Mn increases the formation temperature of α-Fe, retaining the Fe content in the liquid, restricting the stabilization of post-dendritic β-Fe phase. Consequently, to promote the formation of α- Al_15_(Fe,Mn)_4_Si_2_ over β-Al_9_Fe_2_Si_2_ phase, the amount of Mn recommended is above 1.2 wt%.

**Fig. 3:**
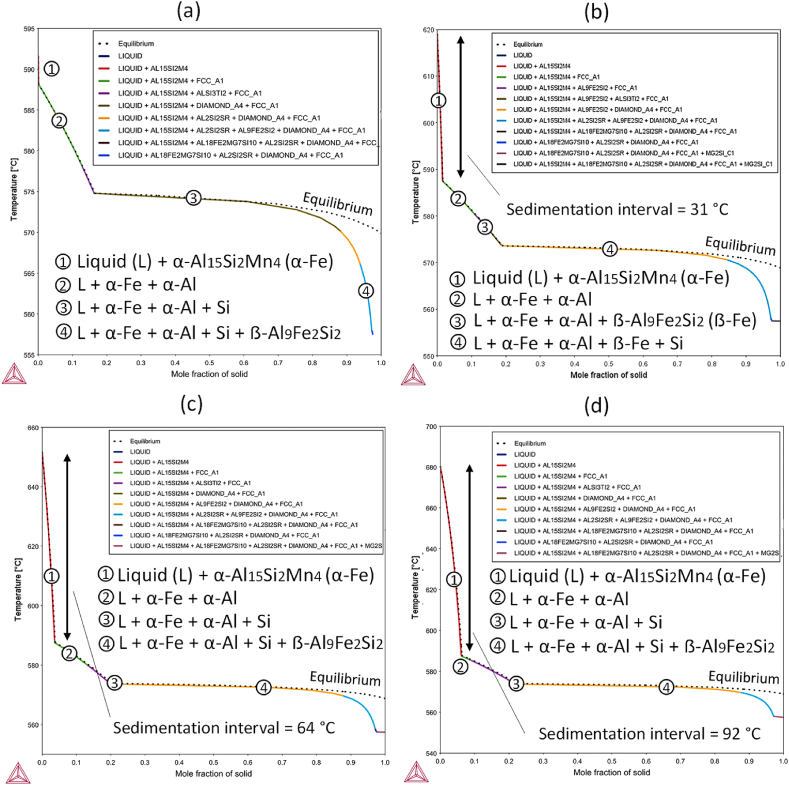
Non-equilibrium solidification of (a) AlSi10MnMg, (b) AlSi10Mn0.7 Mg(Fe), (c) AlSi10Mn1.2 Mg(Fe) and (d) AlSi10Mn2.0 Mg(Fe) alloys obtained by Scheil-Gulliver model.

Based on CALPHAD results, a Mn contents of 0.7 wt%, 1.2 wt% and 2.0 wt%. were selected as target compositions for the experimental alloys. Hereafter the alloys are referred as AlSi10Mn0.7 Mg(Fe), AlSi10Mn1.2 Mg(Fe) and AlSi10Mn2.0 Mg(Fe), respectively. The goal of the selected compositions was to avoid the formation of β-phase at different solidification conditions, and to enable the sedimentation process at the different pouring temperatures of casting processes, specially at high temperatures. The Scheil-Gulliver model was employed to calculate the non-equilibrium solidification properties of the selected compositions. Additionally, the alloy without Fe addition (AlSi10MnMg) was also considered in the calculations. The results of non-equilibrium calculations are reported in Fig. 3. In general, the equilibrium (dotted lines) and non-equilibrium curves showed similar pattern at high temperatures, or low mole fraction of solid. Nevertheless, the curves deviated from equilibrium calculations at the last stages of solidification. The main transformations that occurred during solidification in AlSi10MnMg, AlSi10Mn1.2 Mg(Fe) and AlSi10Mn2.0 Mg(Fe) alloys were as follows: L → α-Fe, L → α-Fe + α-Al, L → α-Fe + α-Al + Si and L → α-Fe + α-Al + Si + β-Fe. On the other hand, in AlSi10Mn0.7 Mg(Fe) alloy β-Fe phase precipitated prior to Si.

In Fig. 3 (a) the solidification sequence of standard AlSi10MnMg (0.15 wt% Fe) alloy is shown. The precipitation of α-Al_15_(Fe,Mn)_4_Si_2_ phase started at 591 °C, and α-Al phase at 588 °C. Hence, gravitational sedimentation of Fe-rich particles cannot occur in the alloy without the addition of Fe or Mn. In contrast to the equilibrium calculations (zone marked as standard range in Fig. 1), the β-Al_9_Fe_2_Si_2_ phase precipitated prior to the solidus point, at 566 °C. In Fig. 3 (b) the solidification pattern of AlSi10Mn0.7 Mg(Fe) alloy is shown. The precipitation of α-Al_15_(Fe,Mn)_4_Si_2_ phase started at 619 °C, and β-Al_9_Fe_2_Si_2_ phase precipitated at 574 °C, increasing the sedimentation interval to 31 °C. Comparing to AlSi10MnMg alloy, the addition of 1.1 wt% Fe promoted the precipitation of β-Al_9_Fe_2_Si_2_ phase prior to Si phase. In Fig. 3 (c) the solidification pattern of AlSi10Mn1.2 Mg(Fe) alloy is shown. The main effect of increasing Mn content was the formation of α-Al_15_(Fe,Mn)_4_Si_2_ phase at 651 °C. The sedimentation interval was 64 °C, and the last phase precipitated was β-Fe. Finally, in Fig. 3 (d) the solidification sequence of AlSi10Mn2.0 Mg(Fe) alloy showed the same solidification pattern than the previous one, but the precipitation temperature of α-Al_15_(Fe,Mn)_4_Si_2_ phase increased to 680 °C, increasing the solidification interval to 92 °C. Consequently, the Mn addition resulted in a significant increase of the sedimentation interval. According to the calculations, the gradual addition of Mn can achieve better Fe removal results, because of the highest precipitation temperature of α-Fe rich phase. Furthermore, the efficiency of the sedimentation process can also be improved by promoting the precipitation of Fe-rich compounds at highest temperature and lowest solid fraction of solid [27].

### 2.2 Materials preparation

According to the thermodynamic modelling of the alloys, three experimental alloys containing Fe and different contents of Mn were manufactured by a gravity permanent mold casting process in an air atmosphere. The alloys were prepared in an induction furnace VIP-I (Inductotherm, Rancocas, USA) and an alumina crucible. The AlSi10MnMg alloy (Silafont®-36, Rheinfelden Alloys, Rheinfelden, Germany) was selected as base alloy to guarantee a melting bath where the other elements were dissolved. The elemental composition of the molten bath (prior to elemental modification) was analyzed by optical emission spectrometry (OES) in a SPECTROMAXx (Spectro, Kleve, Germany) analyzer, the results are shown in Table 1. Then, the AlSi10MnMg alloy was alloyed with Fe to obtain the goal composition of recycled cast aluminum alloys. Subsequently, the melt composition was adjusted to obtain 0.70 wt%, 1.20 wt% and 2.00 wt% of Mn. Tablets of Al–Fe and Al–Mn containing 75 wt% of Fe and 80 wt% of Mn (Bostlan, Mungia, Spain) were used to obtain the goal compositions. The chemical composition in wt.% of the alloys obtained by OES is also shown in Table 1. Finally, the melt was poured at a temperature around 680 °C into a water refrigerated cylindrical mold, and into a preheated permanent steel mold. Approximately, 4.5 kg were obtained for the studied alloys in each mold.

**Table 1:** Elemental composition of AlSi10MnMg(Fe) alloys with different amounts of Mn obtained by OES (wt. %).

Alloy	Al	Si	Fe	Cu	Mn	Mg	Zn	Ti	Others
AlSi10MnMg	Bal.	10.86	0.12	0.02	0.63	0.00	0.22	0.04	≤0.15
AlSi10Mn0.7 Mg(Fe)	Bal.	10.12	1.09	<0.01	0.69	0.21	<0.01	0.04	<0.15
AlSi10Mn1.2 Mg(Fe)	Bal.	10.64	0.93	<0.01	1.24	0.20	<0.01	0.04	<0.15
AlSi10Mn2.0 Mg(Fe)	Bal.	10.37	1.09	<0.01	2.00	0.19	<0.01	0.05	<0.15

### 2.3 Solidification experiments

**Fig. 4:**
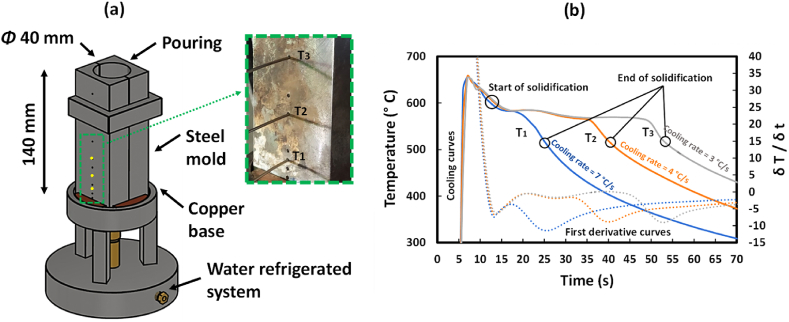
(a) Schematic illustration of the water refrigerated mold with a detailed view of the thermocouples and (b) Solidification path of the AlSi10Mn2.0 Mg(Fe) alloy in the zones defined as T_1_, T_2_ and T_3_.

To study the effects of Mn addition under different CR-s, a purpose-built water refrigerated mold was used. The schematic illustration of the water refrigerated mold is shown in Fig. 4 (a). The data for calculating the CR-s in the denoted T_1_, T_2_ and T_3_ zones were collected using a high-speed National Instruments Data Acquisition System, by introducing K-type thermocouple directly in the melt. The water refrigeration system resulted in a CR gradient in the cylindrical ingot, showing the T_1_ zone the highest CR due to the proximity to the water refrigerated copper base. All solidification experiments were conducted under identical casting conditions. In Fig. 4 (b), the solidification curves and the calculated first derivatives curves of AlSi10Mn2.0 Mg(Fe) alloy in the defined zones (T_1_, T_2_ and T_3_) is plotted as example. The start and the end of solidification of each zone were calculated by differential thermal analysis. The experimental CR-s in the measured zones were T_1_ = 7 °C/s, T_2_ = 4 °C/s and T_3_ = 3 °C/s.

### 2.4 Microstructural characterization

The samples for microstructural characterization were sectioned along the central vertical plane from the ingots solidified in the water refrigerated mold, and prepared according to standard metallographic procedures, by hot mounting in conductive resin, grinding, and polishing. The microstructure was characterized in the different zones where the CR was calculated and, also, in a zone close to the base of the mold, which was expected to show the highest CR, defined as CR > 7 °C/s. The microstructural characterization was performed by means of an optical microscope (OM) model DMI5000 M (LEICA Microsystems, Wetzlar, Germany) and a scanning electron microscope (SEM), equipped with an energy dispersive X-ray spectrometry (EDS) model JSM-5910LV (JEOL, Croissy-sur-Seine, France). The crystallographic structures of Fe-rich intermetallics were determined by means of Electron Backscatter Diffraction (EBSD) scans which were performed on a SEM with a beam voltage of 20 kV and using the EDAX's APEX acquisition software package. The samples for EBSD analysis were prepared using standard metallographic techniques, followed by final polishing with colloidal silica. Finally, the quantitative microstructural characterization of Fe-rich phases and secondary dendrite arm spacings (SDAS) was performed by ImageJ software.

### 2.5 Sedimentation experiments

The ingots casted into preheated steel mold were remelted in graphite crucible in an electric furnace at 710 °C and held for 15 min to reach complete dissolution. Then, the melt was cooled down to their respective maintenance temperature (600 °C and 670 °C) and held at different times (15 and 30 min) before casting. Finally, the samples were taken out of from the furnace to let the melt solidify at room temperature. The temperatures were controlled by introducing K-type thermocouple directly in the center of the melt. Specimens of approximately 80 mm (length) × 55 mm (diameter) were obtained. The samples were sectioned diametrically, grinded, and polished for compositional analysis by OES.

## 3 Results and discussion

### 3.1 Microstructural characterization of the alloys

**Fig. 5:**
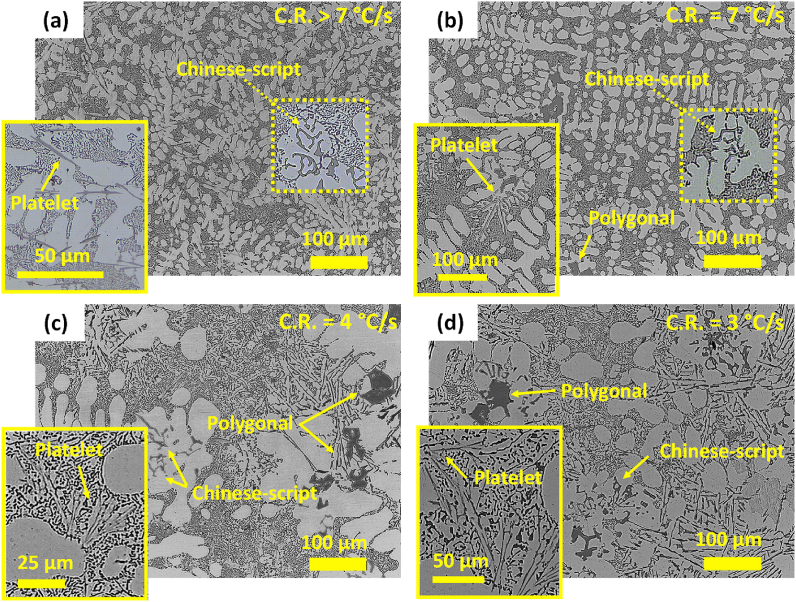
OM images of cast AlSi10Mn0.7 Mg(Fe) alloy solidified at (a) > 7 °C/s, (b) 7 °C/s, (c) 4 °C/s and (d) 3 °C/s.

The OM images of the AlSi10Mn0.7 Mg(Fe) alloy solidified at different CR-s are shown in Fig. 5. In general, the microstructure of the alloy showed a microstructure composed of dendritic α-Al phase, eutectic or near eutectic Si particles, and Fe-rich compounds with different morphologies. In Fig. 5 (a), a predendritic platelet-type morphology and minor Chinese-script morphologies are distinguished at CR > 7 °C/s. These phases were previously defined as β-Fe and α-Fe phase [15], respectively. In Fig. 5 (b), when the CR was decreased to 7 °C/s, a net-like eutectic structure was formed surrounding the cellular and globular-type α-Al dendrites. At this CR, the length and volume of platelet-type morphologies were apparently decreased. The Fe-rich compounds mainly precipitated in a post-dendritic Chinese-script and predendritic polygonal morphologies. The polygonal compounds were assumed to be primary α-Fe or “sludge”. Likely, the lower CR promoted the formation of primary α-Fe resulting in a lower Fe content available for the formation of platelet-type phase. In Fig. 5 (c), the microstructure showed a mixture of coral-like and plate-like Si particles at CR = 4 °C/s. The α-Al dendrites and the eutectic Si were noticeably larger than in previously observed microstructures. The Fe-rich compounds showed a mixture of Chinese-script, star-like, platelet-like, and polygonal morphologies. The platelet-like phase precipitated in the interdendritic space, which means that is a post-dendritic phase. Finally, in Fig. 5 (d) the microstructure solidified at 3 °C/s was like previous one.

**Fig. 6:**
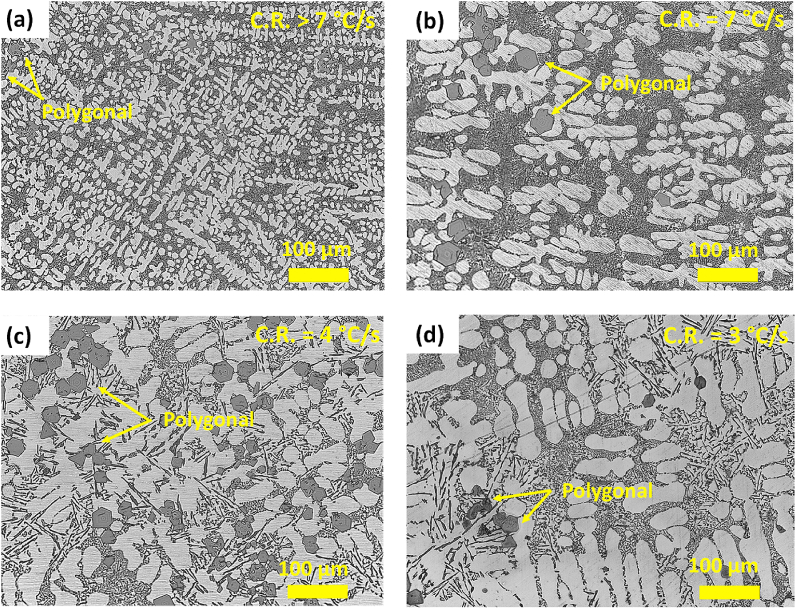
OM images of cast AlSi10Mn1.2 Mg(Fe) alloy solidified at (a) > 7 °C/s, (b) 7 °C/s, (c) 4 °C/s and (d) 3 °C/s.

The OM images of the AlSi10Mn1.2 Mg(Fe) alloy solidified at different CR-s are shown in Fig. 6. In general, the microstructure of the alloy showed a microstructure composed of dendritic α-Al phase, eutectic or near eutectic Si particles, and polygonal Fe-rich compounds. In contrast to the AlSi10Mn0.7 Mg(Fe) alloy, platelet-type or Chinese-script compounds were not observed. In Fig. 6 (a), the microstructure showed fine α-Al dendrites and well dispersed irregular polygonal compounds. In Fig. 6 (b), when the CR was 7 °C/s a high coarsening of the microstructure and Fe-rich compounds with polygonal morphology was observed. In Fig. 6(c and d) at CR-s of 4 °C/s and 3 °C/s respectively, the major effect of decreasing the CR was the transition of the fine eutectic Si to coarse plate-like morphology in some minor interdendritic regions.

**Fig. 7:**
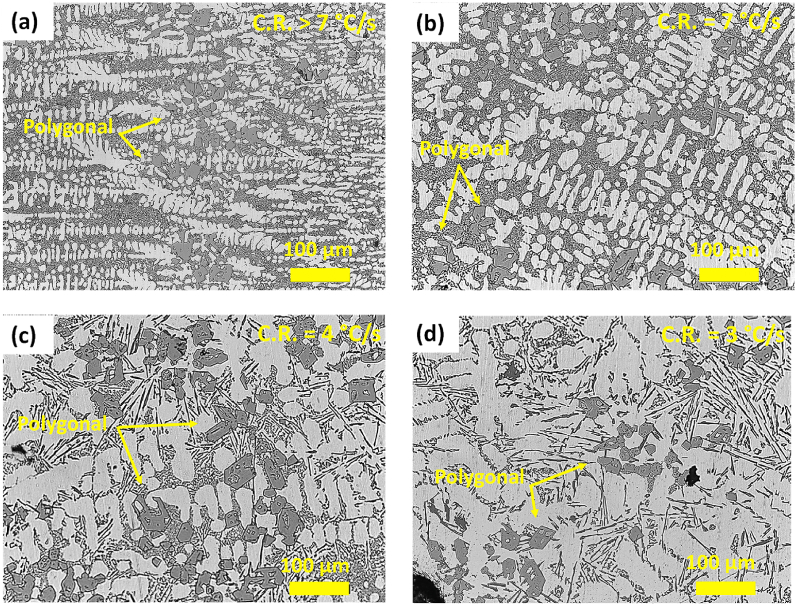
OM images of cast AlSi10Mn2.0 Mg(Fe) alloy solidified at (a) > 7 °C/s, (b) 7 °C/s, (c) 4 °C/s and (d) 3 °C/s.

The OM images of the AlSi10Mn2.0 Mg(Fe) alloy solidified at different CR-s are shown in Fig. 7. As in the previous alloy, the microstructure of the alloy also showed a microstructure composed of dendritic α-Al phase, eutectic or near eutectic Si particles, and polygonal Fe-rich compounds. In Fig. 7 (a), at CR > 7 °C/s the Fe-rich compounds precipitated in polygonal compounds. In Fig. 7 (b), at CR of 7 °C/s there was a generalized coarsening of the microstructure, with Fe forming irregular polygonal phases. In Fig. 7(c and d) at CR-s = 4 - 3 °C/s, the major effect of decreasing the CR was the modification of Si particles from fine choral-like to coarse platelet-like morphology. This also was observed in the alloy containing 1.2 wt% Mn, but the regions with platelet-like Si particles were noticeably higher, especially at C.R = 3 °C/s.

**Fig. 8:**
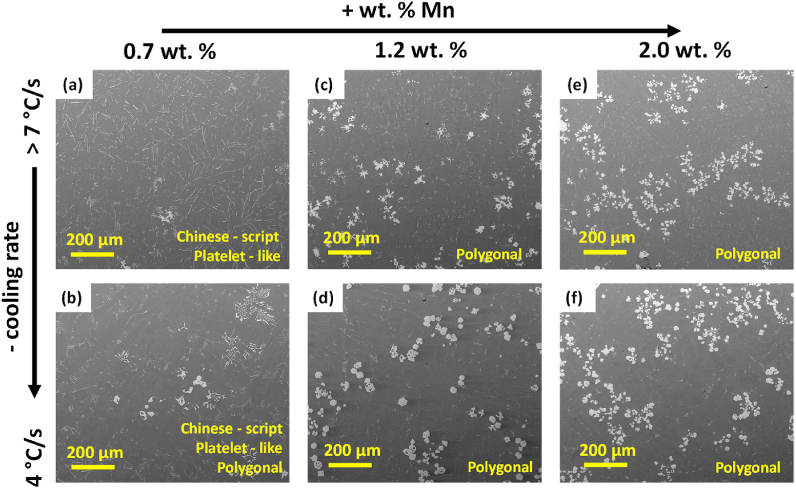
*Backscattered SEM images showing the morphology of Fe-rich compounds solidified at >* 7 °C/s *(first row) and* 4 °C/s *(second row), in (a)–(b) AlSi10Mn0.*7 Mg*(Fe); (c)–(d) AlSi10Mn1.*2 Mg*(Fe) and (e)–(f) AlSi10Mn2.*0 Mg*(Fe) alloys.*

In Fig. 8 backscattered SEM images of Fe-rich compounds comparing different CR-s and Mn contents in the studied alloys are shown. The CR > 7 °C/s (the highest) and CR = 4 °C/s (typical in die casting) were selected of interest. Fig. 8 (a) shows that in AlSi10Mn0.7 Mg(Fe) solidified at > 7 °C/s, a uniformly distributed large platelet-like compound was the major Fe-rich phase, existing also a Chinese-script minor phase. According to the results shown in Fig. 8 (b), the main effect of decreasing the CR was the reduction of detrimental platelet-like phases. The presence of a larger number of Chinese-script compounds and, also, irregular polygonal compounds were confirmed. According to the non-equilibrium calculations in Fig. 3 (b), platelet-like β-phase precipitated prior to Si, so it was impossible to avoid its stabilization in AlSi10Mn0.7 Mg(Fe). In AlSi10Mn1.2 Mg(Fe) alloy, the SEM images showed that platelet-like compounds were not stabilized. Fig. 8 (c) shows that the addition of 1.2 wt% Mn promoted the formation of irregular polygonal compounds at high CR. In the same alloy solidified at 4 °C/s (Fig. 8 d), the precipitated Fe-rich compounds were more regular polygonal shaped, some of them presented holes in the center. Therefore, the formation of β-phase was avoided by increasing the Mn content from 0.7 to 1.2 wt%, which agrees with previous studies [12].

Fig. 8 (e) shows that in AlSi10Mn2.0 Mg(Fe) alloy solidified at CR > 7 °C/s, the Fe-rich compounds exhibited polygonal morphology. Fig. 8 (f) shows that the main effect of decreasing the CR, was the transformation of α-Fe phase from an irregular to a more regular polygonal morphology. Evidently, the further addition of Mn up to 2.0 wt% promoted a higher amount of α-Fe phase.

**Fig. 9:**
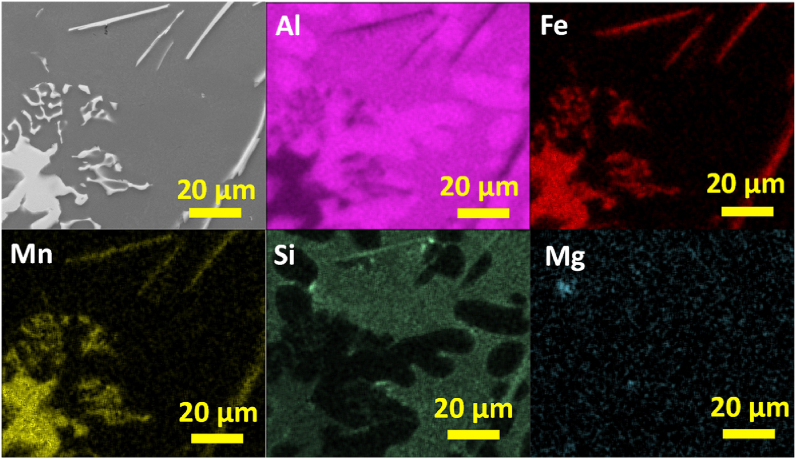
SEM image and EDS elemental mapping of Chinese-script compounds in AlSi10Mn0.7 Mg(Fe) alloy solidified at > 7 °C/s.

To understand the elemental distribution in the previously observed Fe-rich phases, a SEM - EDS qualitative and semiquantitative analysis were conducted. In Fig. 9 the SEM image and EDS elemental mapping of Chinese-script compounds in AlSi10Mn0.7 Mg(Fe) alloy solidified at > 7 °C/s is shown. As can be seen from the elemental maps, the Chinese-script compounds was composed of Al, Fe, Mn, and Si. All the elements displayed uniform distributions within this phase. Whereas Si also was distributed throughout the matrix, as fine and well dispersed fine Si particles. Finally, Mg which was a minor element in the studied alloys, appeared as small particles uniformly dispersed throughout the microstructure.

**Fig. 10:**
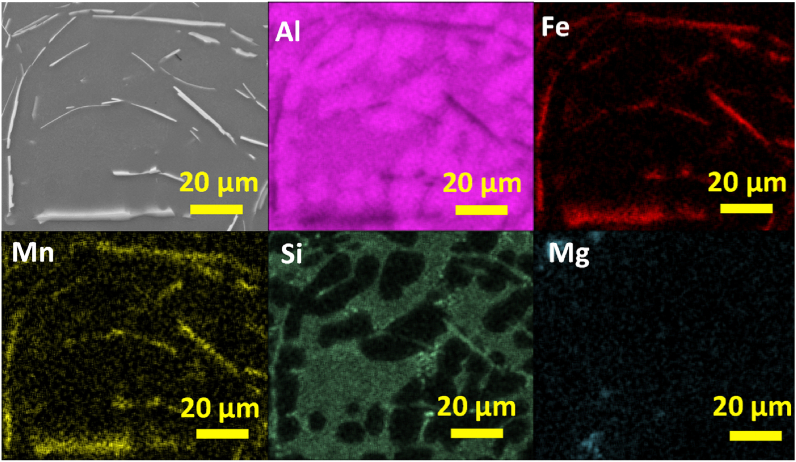
SEM image and EDS elemental mapping of platelet-like compounds in AlSi10Mn0.7 Mg(Fe) alloy at > 7 °C/s.

In Fig. 10 the elemental composition of platelet-like compounds in AlSi10Mn0.7 Mg(Fe) alloy solidified at > 7 °C/s is shown. The platelet-like compounds also showed uniform distribution of Al, Fe, Mn, and Si. Si also was distributed throughout the matrix as fine and well dispersed Si particles, and Mg also appeared as uniformly dispersed particles throughout the microstructure.

**Fig. 11:**
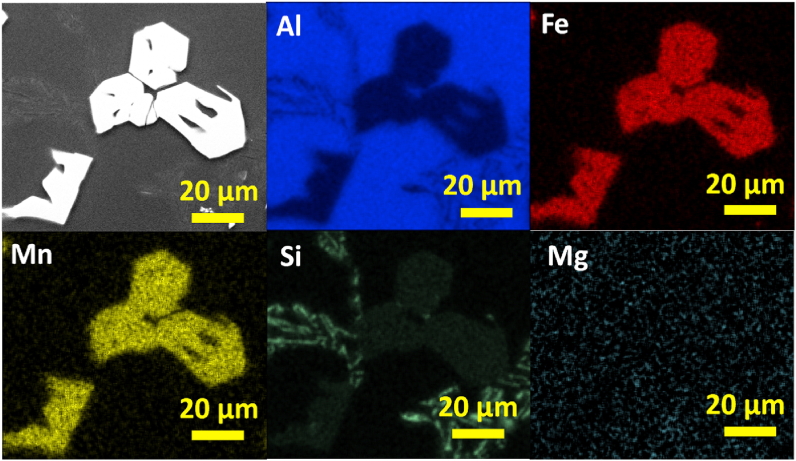
SEM image and EDS elemental mapping of polygonal compounds in AlSi10Mn0.7 Mg(Fe) alloy solidified at 4 °C/s.

In Fig. 11 the elemental composition of polygonal compounds in AlSi10Mn0.7 Mg(Fe) alloy solidified at 4 °C/s is shown. The polygonal phase was mainly composed by Al, Fe, Mn, and Si. The coarsening of Si particles was observed in the elemental mapping since Si also appeared in a bright region forming coarse particles. This was not observed in the previously studied EDS mappings. Finally, uniformly dispersed particles of Mg also were observed.

**Fig. 12:**
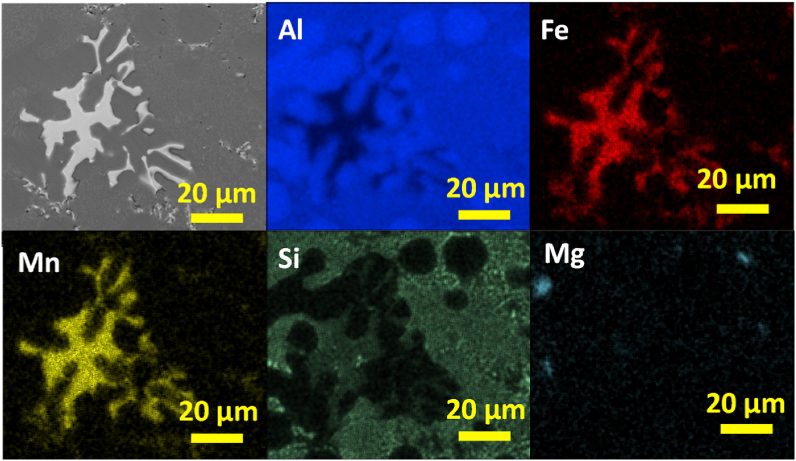
SEM image and EDS elemental mapping of irregular polygonal compound in AlSi10Mn1.2 Mg(Fe) alloy solidified at > 7 °C/s.

In Fig. 12 the SEM image and EDS elemental mapping of irregular polygonal compound in AlSi10Mn1.2 Mg(Fe) alloy solidified at > 7 °C/s is shown. The phase showed a qualitative uniform composition of Al, Fe, Mn, and Si. Whereas Si was also distributed throughout the matrix as fine and well dispersed Si particles. This confirmed that the coarsening of Si particles was not occurred in AlSi10Mn1.2 Mg(Fe) alloy. There were minor Mg-rich regions in the microstructure, but these Mg-rich regions could not be correlated with the formation of Fe-rich phases.

**Fig. 13:**
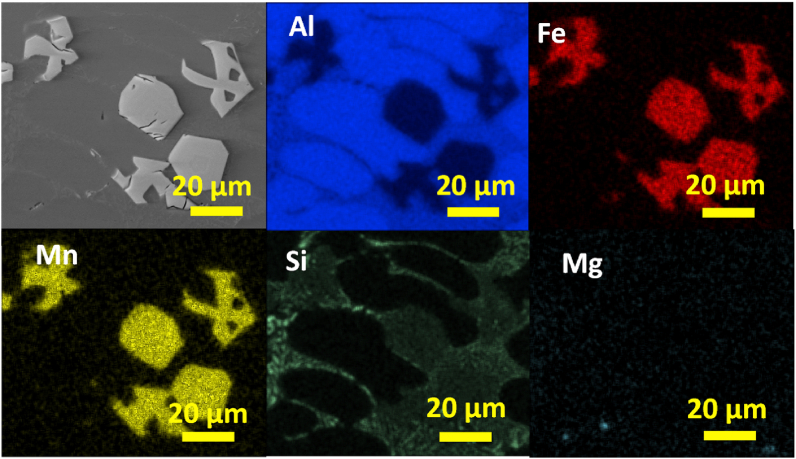
SEM image and EDS elemental mapping of regular polygonal compounds in AlSi10Mn1.2 Mg(Fe) alloy solidified at 4 °C/s.

In Fig. 13 the elemental composition of regular polygonal compounds in AlSi10Mn1.2 Mg(Fe) alloy solidified at 4 °C/s is shown. The regular α-Fe phase was composed of a mixture of Al, Fe, Mn, and Si. There are another two regions composed of Si. In the first one, it showed a fine eutectic morphology particle. In the second one, with the brightest contrast, a coarse plate-like eutectic region was distinguished.

**Fig. 14:**
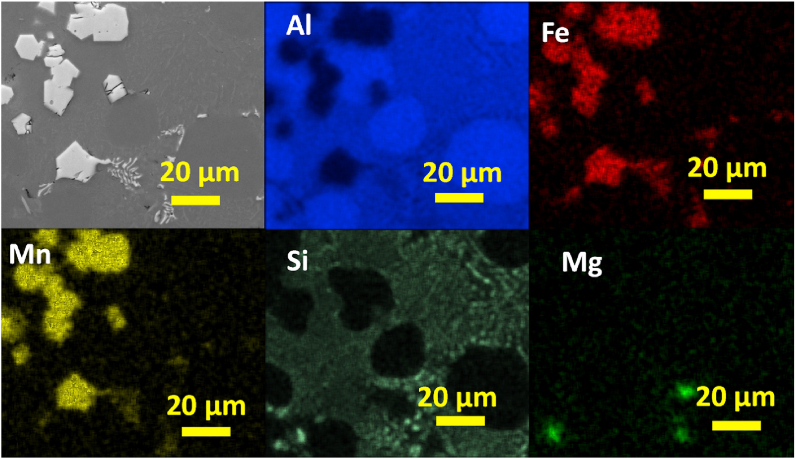
SEM image and EDS elemental mapping of polygonal compounds in AlSi10Mn2.0 Mg(Fe) alloy solidified at 4 °C/s.

In Fig. 14 the SEM image and EDS elemental mapping of polygonal compounds in AlSi10Mn2.0 Mg(Fe) alloy solidified at 4 °C/s is shown. According to the results, Mn and Fe were segregated in the polygonal compounds. The mixture of fine and plate-like Si-rich regions also were observed. Finally, there were minor Mg-rich regions in the microstructure.

The semiquantitative chemical composition of the analyzed Fe-rich phases obtained by EDS is presented in Table 2. The α-Fe phase in the alloys containing 1.2 wt% and 2.0 wt% of Mn showed the same semiquantitative chemical composition. According to the results, β-Fe phase was the phase with the lowest content in Mn, and the highest content in Si. This was observed in previous studies in Al-11.5Si-0.4 Mg [39] and Al–12.7Si alloys [46]. As can be seen in Table 2, β-Fe phase presented the same chemical composition regardless of the CR. Even though, it was precipitated as predendritic compound at CR > 7 °C/s, and as postdendritic at CR = 4 °C/s. In the same alloy, the Chinese-script or postdendritic α-Fe phase showed a higher concentration in Mn. On the other hand, Si was reduced by almost half. The higher Mn content in polygonal compounds was previously reported [25,34]. As can be analyzed from the data, the Mn:Fe and (Mn:Fe):Si mole ratios of β-Fe phase were lower than those of α-Fe. Regarding the α-Fe phase, the Mn:Fe ratio of postdendritic Chinese-script and predendritic polygonal compounds were similar, but the (Mn:Fe):Si mole ratio of Chinese-script compound was lower. This is related to the previously observed phase diagrams in Section 2.1. Since increasing Mn content stabilized the Fe-rich phases at higher temperatures, the Mn:Fe and (Mn:Fe):Si mole ratios of each phase, gradually decreased with the precipitation temperature (Fig. 3) of each phase.

**Table 2:** Semiquantitative chemical composition of Fe-rich intermetallics obtained by EDS (at. %).

Alloy	Phase	CR (°C/s)	Element in at. %				Mn:Fe	(Mn:Fe):Si
			Al	Fe	Mn	Si		
AlSi10Mn0.7 Mg(Fe)	β-Fe	>7	74	5	2	19	0.4	0.4
	α-Fe*		82	4	4	10	1	0.8
	β-Fe	4	76	5	1	18	0.2	0.3
	α-Fe		77	7	6	9	0.9	1.4
AlSi10Mn1.2 Mg(Fe)/AlSi10Mn2.0 Mg(Fe)	α-Fe	>7	77	6	7	10	1.2	1.3
		4	76	6	8	10	1.3	1.4
*Chinese-script								

**Fig. 15:**
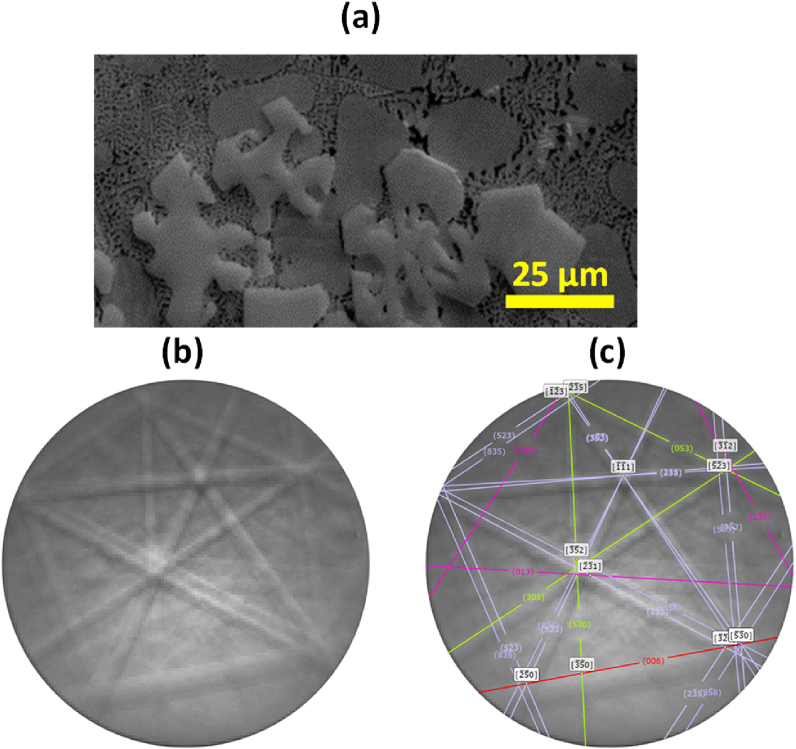
(a) Studied α-Fe intermetallics in AlSi10Mn1.2 Mg(Fe), (b) its experimental EBSD pattern and (c) its simulated EBSD pattern.

For more accurate phase identification, in Fig. 15 (a) the crystal structure of the observed Fe-rich compounds was determined by EBSD. The experimental EBSD patterns (Fig. 15 b) of α-Fe compounds with standard EBSD patterns (Fig. 15 c) of corresponding phases were compared. In this work, all the crystallographic parameters reviewed by Liu et al. were considered for phase identification [25]. As an example, Fig. 15 shows the EBSD patterns results of α-Fe intermetallics in AlSi10Mn1.2 Mg(Fe) alloy. The best matching results were obtained with the standard pattern of the Al_4.01_MnSi_0.74_-type cubic crystal structure (m $\bar{3}$) with a lattice constant of a = 1.264 nm. Therefore, the obtained results agree with the authors that characterized the α-Fe phases as Bcc structure [25,[51], [52], [53]].

### 3.2 Quantitative image analysis of the microstructure

The results of the quantitative analysis of the SDAS and the size of Fe-rich phases obtained by image analysis are summarized in Table 3. According to the experimental results, SDAS values gradually decreased with increasing Mn content and CR. Nevertheless, the CR had a greater influence in the refinement of SDAS than the addition of Mn. Some authors also reported that as the Fe and Mn content increased, the dendritic grain structure was refined [54,55,56]. Therefore, in addition to the effects in Fe-rich phase formation already discussed, the addition of Mn also promoted the grain refinement of the alloys.

**Table 3:** Size of Fe-rich phases of the investigated alloys at different CR (μm).

Alloy	Phase	CR > 7 °C/s	CR = 7 °C/s	CR = 4 °C/s	CR = 3 °C/s
AlSi10Mn0.7 Mg(Fe)	SDAS	8 ± 1	18 ± 4	22 ± 5	22 ± 3
	β-Fe	33 ± 13	33 ± 11	18 ± 10	15 ± 6
	α-Fe	44 ± 12 *	58 ± 17	55 ± 13	54 ± 16
AlSi10Mn1.2 Mg(Fe)	SDAS	8 ± 2	17 ± 3	23 ± 4	25 ± 5
	α-Fe	24 ± 7	32 ± 9	30 ± 8	30 ± 6
AlSi10Mn2.0 Mg(Fe)	SDAS	7 ± 1	14 ± 2	18 ± 4	22 ± 5
	α-Fe	25 ± 12	38 ± 13	39 ± 13	37 ± 17
* Only Chinese-script α-Fe compounds					

In Table 3 the circumscribed diameter of α-Fe, and the length of β-Fe phases also were studied by image analysis and correlated with the processing conditions. The Chinese-script and polygonal morphologies were measured together as they are the same α-Fe phase. The quantitative image analysis of the microstructure in AlSi10Mn0.7 Mg(Fe), showed that a CR ≥ 7 °C/s promoted the formation of the longest β-Fe compounds. At CR ≤ 4 °C/s, the length of these compounds was dramatically decreased to 18–15 μm. On the other hand, α-Fe showed the largest size at CR ≤ 7 °C/s. This is because α-Fe phase is a postdendritic phase with Chinese-script morphology and showed less compact morphology than the predendritic polygonal α-Fe phase (Fig. 8).

In general, increasing the Mn content from 1.2 wt% to 2.0 wt%, reduced the size of polygonal α-Fe particles at studied processing conditions. In AlSi10Mn1.2 Mg(Fe) and AlSi10Mn2.0 Mg(Fe) alloys, the α-Fe compounds showed the smallest size at CR > 7 °C/s, and a constant size below this CR. A smaller sludge (α-Fe) particles in the areas of the casting with higher CR was previously observed by Liu et al. [25]. This agrees with the size of α-Fe compounds reported when the CR > 7 °C/s in the alloys containing 1.2–2.0 wt% Mn. Consequently, a CR > 7 °C/s was required to reduce the size of α-Fe compounds in the studied alloys.

### 3.3 Iron removal by sedimentation experiments

According to the thermodynamic modelling of the alloys in Fig. 3, the gradual addition of Mn increases the precipitation temperature of α-Fe phase. Since the sedimentation of Fe-rich particles occurs when the melt is hold between the formation temperature of Fe-rich compounds and α-Al phase, the holding temperatures of 600 °C (∼10 °C above α-Al) and 670 °C (∼10 °C below maximum α-Fe) were selected. The maximum holding time selected was 30 min, which is a reasonable holding time in the different casting processes of aluminum alloys.

In Table 4 the elemental composition of the solidified ingot after sedimentation treatment at 600 °C is summarized*.* The results showed that at holding temperature of 600 °C at 15 min, the Fe content of all the alloys was reduced at least to 0.65 wt%, and Mn content at least to 0.55 wt%. The most efficient results of Fe removal by a holding time of 15 min was obtained by the addition of 2.0 wt% Mn, obtaining a melt with 0.42 wt% of Fe. Increasing the holding time to 30 min, revealed that the Fe removal in AlSi10Mn0.7 Mg(Fe) was only slightly improved by increasing the holding time. On the other hand, it was possible to reduce the Fe content in AlSi10Mn1.2 Mg(Fe) and AlSi10Mn2.0 Mg(Fe) alloys to 0.40 wt%, and Mn content to 0.49 wt%. Which made the efficiency of the process in Fe purification of 64%. Finally, as Si formed Fe-rich compounds the Si content in the melt also was decreased. But the overall Si content was kept over 9.0 wt% in all the conditions, which is the minimum Si content in AlSi10MnMg alloys.

**Table 4:** Elemental composition of the solidified ingot after sedimentation treatment at 600 °C obtained by OES.

Alloy	Fe (wt.%)	Mn (wt.%)	Si (wt.%)	Holding time (min)
AlSi10Mn0.7 Mg(Fe)	0.65	0.41	9.91	15
AlSi10Mn1.2 Mg(Fe)	0.46	0.54	9.51	
AlSi10Mn2.0 Mg(Fe)	0.42	0.55	10.32	
AlSi10Mn0.7 Mg(Fe)	0.62	0.33	9.97	30
AlSi10Mn1.2 Mg(Fe)	0.40	0.49	9.69	
AlSi10Mn2.0 Mg(Fe)	0.40	0.49	9.63	

The elemental composition of the solidified ingot after sedimentation treatment at 670 °C obtained by OES is shown in Table 5. In some cases, the precipitation of Fe-rich compounds can occur from their formation, also known as natural sedimentation [43,44]. To validate the natural sedimentation at 670 °C, the experiments also were conducted without holding time, and were directly poured into the molds. The results obtained without holding time (0 min), showed that in AlSi10Mn0.7 Mg(Fe) the sedimentation process was not efficient, since the Fe content of the alloy was 0.80 wt%. According to the non-equilibrium thermodynamic modelling, this is related to the narrow sedimentation interval of ∼31 °C between the precipitation temperature of α-Fe and α-Al phases. Increasing the Mn content, the precipitation of predendritic α-Fe phase at higher temperatures was promoted, improving the Fe removal efficiency to 0.46 wt% and 0.58 wt%, respectively. Hence, the natural sedimentation occurred during the cooled down to room temperature. In AlSi10Mn1.2 Mg(Fe) and AlSi10Mn2.0 Mg(Fe) alloys, the wide sedimentation intervals of 64 °C and 92 °C respectively, improved the Fe removal efficiency of the process. When the holding time was increased to 15 min, the Fe removal efficiency was improved in AlSi10Mn0.7 Mg(Fe). Finally, after increasing the holding time to 30 min, the Fe content in the experimental alloys was not further reduced. The overall Si content also was kept over the minimum Si content in AlSi10MnMg alloys.

**Table 5:** Elemental composition of the solidified ingot after sedimentation treatment at 670 °C obtained by OES (wt.%).

Alloy	Fe (wt.%)	Mn (wt.%)	Si (wt.%)	Holding time (min)
AlSi10Mn0.7 Mg(Fe)	0.80	0.59	9.50	0
AlSi10Mn1.2 Mg(Fe)	0.46	0.57	9.50	
AlSi10Mn2.0 Mg(Fe)	0.58	0.42	9.69	
AlSi10Mn0.7 Mg(Fe)	0.61	0.44	10.03	15
AlSi10Mn1.2 Mg(Fe)	0.44	0.56	9.60	
AlSi10Mn2.0 Mg(Fe)	0.45	0.44	10.05	
AlSi10Mn0.7 Mg(Fe)	0.60	0.40	10.02	30
AlSi10Mn1.2 Mg(Fe)	0.43	0.51	9.93	
AlSi10Mn2.0 Mg(Fe)	0.47	0.68	9.60	

**Fig. 16:**
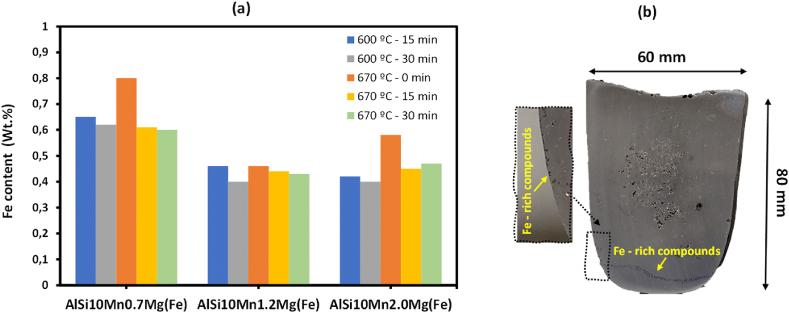
(a) Fe content in the investigated alloys after sedimentation process and (b) cross section of the solidified ingot.

In Fig. 16 (a) the Fe content in the investigated alloys after sedimentation process at different holding temperature and time conditions is plotted. According to the results of the sedimentation treatment at 600 °C, the Fe removal was increased after increasing the Mn content to 1.2 wt%. The further addition of Mn up to 2.0 wt%, showed similar Fe removal values. Regarding the holding times, a high efficiency in Fe removal was achieved in the alloys containing 1.2 wt% and 2.0 wt% of Mn at holding time of 15 min. It was supposed that the liquid was the unique stable phase at 670 °C in AlSi10Mn0.7 Mg(Fe) and AlSi10Mn1.2 Mg(Fe) alloys, so an improvement in the sedimentation process was not expected. The improvement may be since the temperature in the melt was not homogeneously distributed. Therefore, it is assumed that the temperature in the part in contact with the mold is lower, which caused the precipitation and then, the sedimentation of Fe-rich intermetallics. This can be seen in the cross section of the solidified ingot in Fig. 16 (b), where coarse Fe-rich intermetallic compounds were distinguished near the part in contact with the mold wall.

## 4 Conclusions

In this work, CALPHAD approach was employed to study the effects of CR and holding temperature on the modification and purification of iron-rich compounds in AlSi10MnMg(Fe) alloy. The elemental modification was done by addition of Mn, and the microstructure was studied at different CR. To validate the feasibility of the methodology in different processing conditions, the sedimentation experiments were conducted at different holding temperatures and times.

The application of CALPHAD based tools demonstrated that modelling the Fe-rich phase formation as a function of elemental addition and different CR, the microstructure of aluminum castings can be controlled to improve the quality of secondary aluminum alloys. According to the non-equilibrium calculations, the formation of detrimental β-Fe phase cannot be avoided in AlSi10Mn0.7 Mg(Fe) alloy under studied CR. The formation of α-Fe rich phases with Chinese-script and polygonal morphologies were promoted by decreasing the CR, but the formation of β-Fe compounds were not completely inhibited. Two different precipitation sequences of β-Fe phase were observed in AlSi10Mn0.7 Mg(Fe) alloy. In the first one, at CR ≥ 7 °C/s, a predendritic platelet-like β-phase with a length of 33 μm was precipitated in the dendritic region. In the second one, at CR = 3–4 °C/s, the β-phase precipitated as post-dendritic compound with a length of 15–18 μm in the interdendritic region.

Increasing the Mn content from 0.7 wt% to 2.0 wt % promoted the stabilization of predendritic α-Fe phase, avoiding the stabilization of β-Fe phase. According to the non-equilibrium calculations, β-Fe phase precipitated from the liquid in the last stage of solidification, but the stabilization of Fe in predendritic compounds at higher temperatures inhibited the formation of the phase. A clear influence of the CR on the size and morphology of Fe-rich compounds was observed. A CR higher than 7 °C/s was required to reduce the size of polygonal α-Fe compounds. In general, the increase of Mn and a decrease in the CR, promoted the formation of a coarser Fe-rich compounds.

In the sedimentation experiments conducted at 600 °C, the results showed a high Fe removal efficiency up to 64% after holding time of 15 min. In the sedimentation experiments conducted at 670 °C, the results showed a high Fe removal efficiency up to 61% after holding time of 30 min. In general, as the Mn content was increased the Fe removal efficiency was improved.

In the view of the results obtained, an exhaustive study of the tensile properties of as-cast and heat treated AlSi10Mn1.2 Mg and AlSi10Mn2.0 Mg alloys with at least 1.1 wt % Fe is proposed as further work. The alloy should be manufactured by large scale casting process and solidified at different CR after a holding time of 30 min at ≥ 670 °C prior to casting.

## Author contribution statement

Jon Mikel Sanchez, Maribel Arribas: Conceived and designed the experiments; Performed the experiments; Analyzed and interpreted the data; Contributed reagents, materials, analysis tools or data; Wrote the paper.

Haize Galarraga, Maider Garcia de Cortazar, Marco Ellero, Franck Girot: Conceived and designed the experiments; Analyzed and interpreted the data; Contributed reagents, materials, analysis tools or data; Wrote the paper.

## Funding statement

This work was supported by the Basque Government through the projects Elkartek CIRCU-AL: KK-2020/00016 and SosIAMet KK-2022/00110.

## Data availability statement

Data included in article/supplementary material/referenced in article.

## Declaration of interest′s statement

The authors declare no conflict of interest.

## Additional information

No additional information is available for this paper.
